# Prevalence and determinants of gestational weight gain among pregnant women in Niger

**DOI:** 10.1111/mcn.12887

**Published:** 2019-09-30

**Authors:** Césaire T. Ouédraogo, K. Ryan Wessells, Rebecca R. Young, M. Thierno Faye, Sonja Y. Hess

**Affiliations:** ^1^ Program in International and Community Nutrition, Department of Nutrition University of California Davis California USA; ^2^ Helen Keller International Niamey Niger

**Keywords:** low income countries, maternal nutrition, nutritional status, pregnancy, undernutrition, weight gain

## Abstract

Low gestational weight gain (GWG) and low mid‐upper arm circumference (MUAC) are associated with adverse pregnancy outcomes. We aimed to assess the prevalence and determinants of low GWG and low MUAC among pregnant women in rural Zinder, Niger. A community‐based survey was conducted among 1,384 pregnant women in the catchment areas of 18 integrated health centers in the region of Zinder, Niger. Weight and MUAC were measured during an in‐home visit and again 1 month later, when haemoglobin concentration and micronutrient status were also assessed. The prevalence of low GWG was defined based on the 2009 United States Institute of Medicine (U.S. IOM) guidelines (<0.35 kg/week) and less than the third centile of the International Fetal and Newborn Growth Consortium for the 21st Century (INTERGROWTH‐21st) standards. Factors associated with GWG and MUAC were identified using logistic regression models adjusting for season, village, and gestational age. The median (interquartile range) age was 25.0 (20.7, 30.0) years, and 16.4% were ≤19 years. The prevalence of low GWG were 62.9% and 27.5% according to 2009 IOM and less than the third INTERGROWTH‐21st centile, respectively; 24.9% had low MUAC. Higher α‐1‐acid glycoprotein (OR = 1.7, 95% CI [1.1, 2.8]) and C‐reactive protein (OR = 1.2, 95% CI [1.02, 1.50]) increased the odds of low GWG. Adolescents (OR = 2.7, 95% CI [1.8, 4.0]), housewives (OR = 1.97, 95% CI [1.36, 2.86]), and those who reported recent food assistance (OR = 1.80, 95% CI [1.04, 3.11]) had higher odds of low MUAC. Prevalence of low GWG and low MUAC was high among pregnant women. Determinants of GWG and MUAC included socio‐economic, demographic, and biological factors, although only markers of inflammation were consistent predictors across different definitions of low GWG.

**Key messages**

- GWG and MUAC are strong predictors of birth outcomes. In low‐income countries, information on GWG and MUAC is limited.
- There was a high prevalence of low GWG and low MUAC among pregnant women in rural Zinder, Niger.
- Determinants of GWG and MUAC included socio‐economic, demographic, and biological factors, particularly markers of inflammation.
- Considering the importance of adequate GWG and MUAC for maternal health and pregnancy outcomes, effective and cost‐effective interventions to ensure adequate GWG and nutritional status are needed.

**List of abbreviations**
AGP: α‐1‐acid glycoprotein (AGP)
ANC: Antenatal care
CRP: C‐reactive protein
GA: Gestational age
GEE: Generalized estimated equation
GWG: Gestational Weight Gain
HFIA: Household Food Insecurity Access
HRP2: Histidine‐rich protein 2
IHC: Integrated Health Center
IUGR: Intrauterine growth restriction
KAP: Knowledge Attitude and Practice
LBW: Low birth weight
MDD‐W: Minimum Dietary Diversity for Women
MUAC: Mid‐Upper Arm Circumference
NiMaNu: Niger Maternal Nutrition
OR: Odds ratio
pZn: Plasma zinc
RBP: Retinol binding protein
SES: Socio‐economic status
SFH: Symphysis fundal height
SGA: Small for gestational age
sTfR: Soluble transferrin receptor
U.S. IOM: United States Institute of Medicine
WHO: World Health Organization

## 1 INTRODUCTION

Gestational weight gain (GWG) is a complex biological phenomenon that influences pregnancy outcomes (IOM and NRC, 2009). An appropriate GWG is essential for optimal pregnancy outcomes for both the mother and her infant (Asefa & Nemomsa, 2016). Low GWG is associated with adverse outcomes including low birth weight (LBW; Edwards, Hellerstedt, Alton, Story, & Himes, 1996; Hellerstedt, Himes, Story, Alton, & Edwards, 1997; Hickey, McNeal, Menefee, & Ivey, 1997; Schieve, Cogswell, & Scanlon, 1998), intrauterine growth restriction (Strauss & Dietz, 1999), small for gestational age (SGA; Hellerstedt et al., 1997; Hickey et al., 1997; Nielsen, Gittelsohn, Anliker, & O'Brien, 2006), and preterm delivery (Hickey, Cliver, Goldenberg, McNeal, & Hoffman, 1995; Stotland, Cheng, Hopkins, & Caughey, 2006). Excessive GWG is associated with an increased risk for macrosomia, gestational diabetes (Hedderson et al., 2006), cesarean section, and postpartum weight retention (Caulfield, Stoltzfus, & Witter, 1998; Hellerstedt et al., 1997; Viswanathan et al., 2008). Determinants of GWG include a range of maternal biological (e.g., age, parity, stature, and genetics), metabolic, and social factors (e.g., socio‐economic status [SES], education, physical activity, and diet; IOM and NRC, 2009).

In its 2009 guidelines, the U.S. Institute of Medicine (IOM) recommended a range for total GWG of 5 to 18 kg, with a GWG rate of 0.17 to 0.58 kg per week during the second and third trimesters (IOM and NRC, 2009). These guidelines of GWG are based on pre‐pregnancy body mass index (BMI; IOM and NRC, 2009) and may only be relevant for women in the United States and other high‐income countries. For low‐income countries across the world, there has been limited information on recommended GWG, and the World Health Organization (WHO) does not currently have recommendations for GWG. However, the International Fetal and Newborn Growth Consortium for the 21st Century Project (INTERGROWTH‐21st), which was implemented in eight countries, has recently published GWG standards based on gestational age (GA) in weeks (Cheikh Ismail et al., 2016). These standards describe the GWG patterns of normal weight women at low risk of adverse maternal and perinatal outcomes and are intended to be applied globally.

Mid‐upper arm circumference (MUAC) is also a strong indicator for predicting adverse birth outcomes in low‐resource settings (Ververs, Antierens, Sackl, Staderini, & Captier, 2013). Low‐maternal MUAC was shown to be associated with an increased risk of LBW (Assefa, Berhane, & Worku, 2012; Karim & Mascie‐Taylor, 1997; Lechtig, 1988; Mohanty et al., 2006; Rollins et al., 2007; Sebayang et al., 2012), preterm labour/birth (Begum, Buckshe, & Pande, 2003; Kalanda, Verhoeff, Chimsuku, Harper, & Brabin, 2006; Sebayang et al., 2012), disproportionate intrauterine growth (Kalanda et al., 2006), birth asphyxia (Lee et al., 2009), and SGA (Sebayang et al., 2012).

Due to the lack of WHO GWG recommendations, and a shortage of basic supplies, including scales, in many health centers in low‐income countries, GWG monitoring is not a common practice in many parts of the world. Moreover, it has been shown that health workers lack adequate knowledge and skills to effectively monitor GWG (Goiburu, Alfonzo, Aranda, Riveros, & Ughelli, 2006; Mowe et al., 2006; Mowe et al., 2008). In Niger, the preterm birth rate and LBW prevalence are high, with 9.4 preterm births per 100 live births (Blencowe et al., 2012) and 27% of infants born LBW (UNICEF, 2017). In addition, the fertility rate of 7.4 children per woman is the highest in the world (UNICEF, 2017). However, information on pregnant women's nutritional status and GWG are limited. Thus, the objectives of the present study were to estimate the prevalence and the determinants of low GWG and low MUAC among pregnant women in Zinder, Niger.

## 2 METHODS

### 2.1 Study design and participants

The present study was a community‐based survey conducted as part of the baseline assessment for the Niger Maternal Nutrition (NiMaNu) Project. This project was a programmatic intervention to improve antenatal care (ANC) services and compared pre‐ and post‐intervention cohorts of pregnant women. Methods for the baseline survey assessments have been described in detail elsewhere (K. Begum et al., 2018; Wessells et al., 2017). Briefly, the community‐based baseline survey was conducted from March 2014 to September 2015. We enrolled pregnant women in 68 rural villages belonging to 18 integrated health centers (IHCs) in the catchment area of two health districts (Mirriah and Zinder), in the Zinder region, Niger. The 18 IHCs were selected based on their accessibility and their distance to Zinder, the regional capital, and because of the limited number and scope of interventions that were being implemented in their catchment area. Within the catchment area of each IHC, the village containing the IHC was automatically included in the survey, as well as one additional randomly selected village among those with a health post. Among the remaining villages in the catchment area of each IHC, four villages ≤10 km and four villages >10 km were randomly selected and randomized to order of participation. Pregnant women from the first two selected villages (IHC‐village and health post village), and the first two villages from the subsequent randomization were enrolled, with a target of enrolling approximately 16–20 women per village and a sample size of approximately 77 women per IHC. When the target number of women was not met by the first four villages in each IHC, women were included from the remaining villages of each IHC following the order of the randomization list. Within each village, participants were identified using a random walk method (United Nations, 2008).

The enrolment of participants was implemented continuously over a period of 18 months with approximatively one new IHC surveyed each month. All identified pregnant women (regardless of gestational week) were eligible for study participation, if they had resided in a participating village for at least 6 months prior to enrolment and had no plans to move out of the study area within the next 2 months. A woman was excluded if she had a severe illness warranting immediate hospital referral or was unable to provide consent due to impaired decision‐making ability.

### 2.2 Data collection and outcomes

Each enrolled woman participated in two study visits. During the first contact (Visit 1), we obtained written informed consent and interviewed women using a structured questionnaire to collect information regarding SES, demographics, and knowledge, attitude, and practices relating to diet, health, pregnancy (current and previous), and ANC attendance. Pregnant women were weighed in light clothing in duplicate to 50‐g precision (SECA 874). Women's height (SECA 213, Seca, Hamburg, Germany), MUAC (ShorrTape© Measuring Tape), and symphysis‐fundal height (ShorrTape© Measuring Tape, Weigh and Measure, Olney, MD) were measured in duplicate to 0.1‐cm precision. A third measurement was performed, and the mean of the two closest measurements was calculated when the first two measurements were >0.2 kg (weight) or >0.5 cm apart (height, MUAC, and symphysis‐fundal height).

Approximatively 1 month later (Visit 2), each participating pregnant woman was invited to a follow‐up assessment. The structured interviews and anthropometric measurements were repeated. Capillary blood samples were drawn to assess haemoglobin concentration by HemoCue® Hb 201+ (Hemocue, Inc; Lake Forest, CA). As described elsewhere (Wessells et al., 2017), venous blood samples (7.5 ml) were collected in a subgroup of participants for the measurement of folate, vitamin B_12_, retinol binding protein, plasma ferritin, soluble transferrin receptor (sTfR), zinc, α‐1‐acid glycoprotein (AGP), C‐reactive protein (CRP), and histidine‐rich protein II (HRP2) concentrations.

GA was estimated as a weighted average of the following obtained information: reported last menstrual period (by estimated number of months, lunar cycles, and/or proximity to a religious or cultural event), time elapsed since quickening, and two fundal height measurements taken approximately 1 month apart (Hess & Ouedraogo, 2016). Three proxy indices (housing quality, household assets, and household livestock) were used to estimate the household SES, as previously described (K. Begum et al., 2018). Household food insecurity was assessed using the Household Food Insecurity Access categories (Coates, Swindale, & Bilinsky, 2007). Pregnant women's dietary practices were assessed using a list‐based food frequency questionnaire, and those who reported consuming at least five of 10 defined food groups in the previous 24 hr were considered to meet the Minimum Dietary Diversity for Women (FAO and FHI 360, 2016).

The outcomes of this study included GWG per week (in kilograms), low GWG, MUAC (in centimeters), and low MUAC. GWG per week was calculated by subtracting weight at Visit 1 from the weight recorded on Visit 2 divided by the number of elapsed days and multiplied by seven. Adequacy of GWG was assessed by comparing GWG of the study participants with the 2009 U.S. IOM guidelines for GWG and the INTERGROWTH‐21st standards, as described in more detail below. Low MUAC was defined as <23 cm (Ververs et al., 2013).

#### 2.2.1 GWG compared with the 2009 U.S. IOM guidelines for GWG

The mean GWG recommended by the U.S. IOM is 0.45 kg/week, with ranges from 0.44 to 0.58 kg/week for underweight and 0.35 to 0.50 kg/week for normal weight women, respectively (IOM and NRC, 2009). These guidelines are based on pre‐pregnancy BMI, which are not known for the participants in the present study. Thus, considering that the majority of women in the present study were likely to be either underweight or of normal weight (Institut National de la Statistique & ICF International, 2007), a GWG of 0.35–0.58 kg/week was considered within the IOM guidelines. Less than 0.35 kg/week was considered as GWG below the IOM guidelines (or a proxy for low GWG) and >0.58 kg/week as GWG above the IOM guidelines (or a proxy of excessive GWG). Because the IOM guidelines apply to women in the second and third trimester of gestation, women in their first trimester of pregnancy were excluded from this classification. Considering that the majority of women had low or adequate GWG in the present study population, GWG was transformed in a dichotomous variable (i.e., GWG < 0.35 kg and GWG ≥ 0.35 kg) to assess factors associated with low GWG.

#### 2.2.2 GWG compared with the INTERGROWTH‐21st standards

We calculated the observed GWG per week for each pregnant woman according to her estimated GA in weeks. Because the published INTERGROWTH‐21st standards represent cumulative GWG, they cannot be directly compared with the observed GWG per week. Rather, the observed GWG was compared with the GWG rate per week of women enrolled in the Fetal Growth Longitudinal Study of the INTERGROWTH‐21st Project (personal communication). Observed GWG less than the third centile of the GWG by GA of the INTERGROWTH‐21st standards was considered to be GWG below the INTERGROWTH‐21st standards (or a proxy for low GWG). Observed GWG between the third and the 97th centile was considered GWG within the standards (or a proxy of normal weight gain), and observed GWG > 97th centile of expected GWG was considered GWG above the INTERGROWTH‐21st standards (or a proxy of excessive GWG). Observed GWG per week of the participants was also compared with the median of the INTERGROWTH‐21st standards and categorically defined as being above or below the INTERGROWTH‐21st median.

### 2.3 Sample size

The overall sample size for the NiMaNu project was specified to be able to detect with 80% power a difference of 10% in the prevalence of anaemia as the primary outcome of the programmatic intervention (Hess & Ouedraogo, 2016). Assuming an initial anaemia prevalence of 50%, a significance level of 0.05, power of 0.80, and a design effect of 2 to account for the cluster sampling design, a sample size of 768 was needed, which was then inflated by 17% for attrition, yielding a target sample size of 925 pregnant women for the baseline survey. However, for the impact assessment of the main trial described elsewhere (Hess & Ouedraogo, 2016), the baseline survey was extended for 6 months to allow statistical models to account for the potential seasonal effects of participants' enrolment on the outcome measures. Based on the same assumption as above, the additional sample size required for the baseline survey was estimated at 77 pregnant women per month for a total of 463 over 6 months. In total, 1,388 pregnant women were needed to be enrolled in the baseline survey to provide 80% power to detect a difference of 10% in the prevalence of anaemia in the primary impact assessment. Data were successfully obtained from 1,385 pregnant women. This sample size was adequate to estimate the prevalence of low GWG + 3.5% (95% CI), assuming a prevalence of 50%.

### 2.4 Statistical analysis

Data were double‐entered and compared using EpiData Entry version 3.1 (Odense, Denmark). All statistical analyses were performed using the SAS System version 9.4 (SAS Institute, Cary, NC, USA). We analysed available data from the baseline survey of the NiMaNu project. Data were examined using univariate analysis (graphical plotting) to look for outliers. Outliers that were clearly impossible or implausible values were corrected if possible, or trimmed when correction was not possible, which was the case for one GWG and one MUAC measurement. A detailed statistical analyses plan is available (Hess & Ouedraogo, 2016).

GWG per week and MUAC were assessed for conformance to the normal distribution. Predictors not normally distributed (i.e., ferritin, sTfR, CRP, AGP, and folate and vitamin B_12_) were natural log transformed. Descriptive analysis of initial characteristics of study participants was performed. Factors associated with low GWG and low MUAC, as well as GWG per week and MUAC as continuous variables, were identified using generalized estimating equation models, in SAS proc glimmix to permit adjusting for cluster effects by village. All models were minimally adjusted to include year, season, and village, and analyses were performed using robust standard errors. All predictors were run in individual models, and the minimally adjusted odds ratio (for low GWG and MUAC as binary outcomes) and the minimally adjusted mean difference (for GWG and MUAC as continuous outcomes) from each individual model were reported.

Potential predictors were identified based on a literature review and background knowledge and prespecified in the statistical analyses plan (Hess & Ouedraogo, 2016). These included maternal age, education, number of pregnancies, number of living children, height, occupation, SES, household food insecurity, reported increase or decrease in the number of meals per day and quantity of food consumed due to pregnancy, reported receipt of food assistance, adequate minimum dietary diversity, micronutrient status (plasma ferritin, zinc, and retinol binding protein adjusted for inflammation; Wessells et al., 2017; sTfR, vitamin B_12_, and folate), markers of inflammation (AGP and CRP), and malaria antigenemia (HRP2). To explore which predictors were consistently and significantly associated with GWG per week and low GWG, we ran five independent analyses including GWG per week and GWG < 0.35 kg/week adjusting for women's GA, and GWG per week, GWG less than the third centile and GWG < 50th centile INTERGROWTH‐21st standards not adjusting for GA following the methods of the respective standards (Cheikh Ismail et al., 2016; Hutcheon & Bodnar, 2018; IOM and NRC, 2009). If a predictor was associated with at least three GWG (GWG per week and/or different definitions of low GWG) or both of the MUAC outcomes (MUAC in centimeters or low MUAC), it was considered to be a consistent predictor of low GWG or undernutrition. A *P* value <.05 was considered as statistically significant for the all tests performed.

### 2.5 Ethics

This study was part of the NiMaNu Project, which was approved by the National Ethical Committee in Niamey (Niger) and the Institutional Review Boards of the University of California, Davis (USA). The study was registered at www.clinicaltrials.gov as NCT01832688. In the presence of a neutral witness, consent materials were presented in both written and oral formats. Informed consent was obtained and documented with a written signature or a fingerprint.

## 3 RESULTS

### 3.1 Characteristics of study population

**Figure 1:**
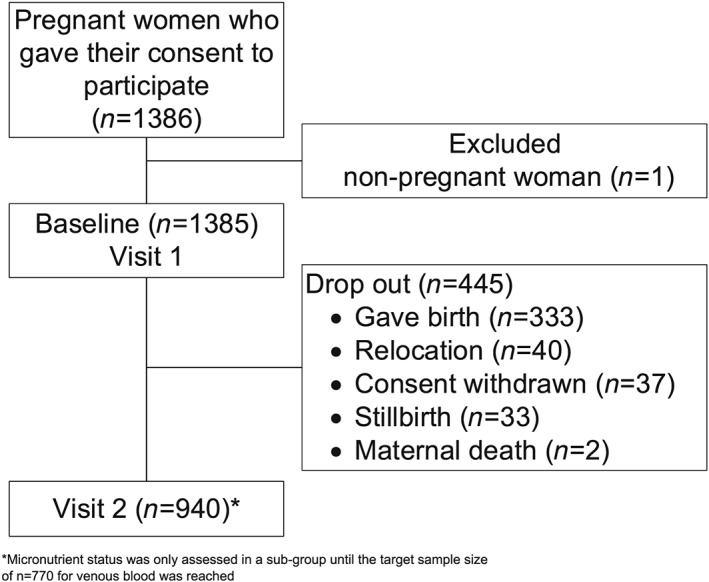
Flow chart for participants included in the present study

A total of 1,385 pregnant women were enrolled during the baseline survey, and 67.9 % (*n* = 940) completed Visit 2 (Figure 1), with 81.9 % (*n* = 770) of these women assessed for micronutrient status. Attrition at Visit 2 was due to birth, relocation, consent withdrawal, stillbirth, and maternal death. The mean participants' age was 26.2 ± 6.4 years, and only 20.9% had attended any formal schooling. The majority of the participants considered themselves to be primarily housewives (83.2%), and 53.1% were in their third trimester of gestation (Table 1). Of the 1,385 pregnant women interviewed, 39.5% of women had attended at least four ANC during their last pregnancy as described in Table 1.

**Table 1:** Characteristics of pregnant women who were enrolled in the baseline survey

Variables	Value
*N*	1,385
Age, years (mean ± *SD*)	26.2 ± 6.4
Adolescent (≤19 years)	221 (16.4%)
Adult (>19 years)	1,131 (83.6%)
Ethnicity	
Hausa	1,188 (85.8%)
Others	196 (14.2%)
Education	
Any formal education	289 (20.9%)
No formal education or literacy training only or koranic school	1,095 (79.1%)
Principal occupation	
Housewife	1,151 (83.2%)
Nonhousewife	233 (16.8%)
Marital status	
Married	1,363 (99.1%)
Separated/divorced or widow	13 (0.9%)
Trimester^a^	
First	33 (2.4%)
Second	612 (44.5%)
Third	730 (53.1%)
Obstetric history	
Age at first pregnancy, median (25th, 75th)	16.0 (16.0–17.0)
(Min–max)	(12.0–35.0)
Gravidity	
Primigravida	178 (12.9%)
Multigravida	1,206 (87.1%)
Number of pregnancies, median (25th, 75th)	5 (3–8)
Number of living children, median (25th, 75th)	3 (2–5)
Outcome of previous pregnancy	
Child born alive, still living	1,029 (85.3 %)
Child not born alive or born alive and had since died	177 (14.7%)
Height (cm)^b^	158.2 (157.9, 158.6)
Attended any ANC during last pregnancy	1,108 (91.9%)
Attended at least four ANC during last pregnancy	476 (39.5%)
Health facility delivery during last pregnancy	419 (34.9%)
Reported food intake during the week before the interview	
Increased	286 (20.7%)
Decreased	797 (57.7%)
No change	298 (21.6%)
Biochemistry assessment^c^	
Haemoglobin concentration, g/dl	9.6 (9.5, 9.7)
Plasma folate concentration, nmol/L	11.2 (10.9, 11.5)
Plasma vitamin B12 concentration, pmol/L	181.3 (176.3, 186.2)
Retinol binding protein concentration,^d^ μmol/L	1.08 (1.06, 1.10)
Plasma ferritin concentration,^d^ μg/L	42.8 (40.0, 45.5)
Soluble transferrin receptor concentration, mg/L	8.69 (8.33, 9.04)
Plasma zinc concentration,^d^ μg/dL	52.2 (51.6, 52.9)
α‐1‐acid glycoprotein concentration, g/L	0.43 (0.41, 0.45)
C‐reactive protein concentration, mg/L	5.32 (4.56, 6.08)
Plasma histidine‐rich protein II concentration, ng/ml	0.17 (0.14, 0.20)
Household level characteristics	
Household head's education level	
Any formal education	261 (21.6%)
No formal education or literacy training only or koranic school	950 (78.4%)
Principal occupation of the household head	
Farming related occupation	578 (42.0%)
Nonfarming‐related occupation	798 (58.0%)
Levels of household food insecurity	
Food secure	432 (31.2%)
Mildly food insecure	150 (10.9%)
Moderately food insecure	345 (25.0%)
Severely food insecure	454 (32.9%)
Season of enrolment	
Lean, rain (June–September)	501 (36.2%)
Dry, postharvest (October–February)	467 (33.7%)
Hot (March–May)	416 (30.1%)

Abbreviations: ANC, antenatal care.

a Trimester at Visit 1.

b Mean (95% CI).

c Sample size varied by indicator: Haemoglobin, *n* = 919; Plasma folate, *n* = 739; Plasma vitamin B_12_, *n* = 744; Retinol binding protein, *n* = 769; Plasma ferritin, *n* = 769; Soluble transferrin receptor, *n* = 769; Plasma zinc, *n* = 723; α‐1‐acid glycoprotein, *n* = 769; C‐reactive protein, *n* = 769; Plasma histidine‐rich protein II, *n* = 769.

d Adjusted for acute phase proteins

GWG analyses included all pregnant women who completed follow‐up Visit 2 and were in the second and third trimester of pregnancy (*n* = 917); 1,384 women enrolled at Visit 1 were included in the MUAC analyses. Using the U.S. IOM 2009 guidelines and the INTERGROWTH‐21st standards, the prevalence of low GWG was 62.9% and 27.5%, respectively (Table 2). In contrast, 13.1% and 2.0% of pregnant women were above the 2009 IOM guidelines and above the 97th centile of the INTERGROWTH‐21st standards, respectively, indicating excessive GWG (Figure S1, Table 2). The median MUAC was 24.1 (interquartile range 18.0, 36.9) cm, and 24.9% had low MUAC (Table 2).

**Table 2:** Prevalence of low GWG, excessive GWG, and low MUAC among study participants

Variables	Value
Participants (*n*)^a^	1,385
GWG per week, kg, in second trimester^b^	0.27 (−2.01, 3.05)
GWG per week, kg, in the third trimester^b^	0.20 (−2.29, 2.96**)**
Classification of GWG	
According to the IOM guidelines	
GWG below the IOM guideline for GWG	574 (62.9%)
GWG within the IOM guideline for GWG	218 (23.9%)
GWG above the IOM guidelines for GWG	119 (13.1%)
According to the INTERGROWTH‐21st standards	
GWG less than the third centile	252 (27.5%)
GWG <50th centile	758 (82.7%)
GWG >97th centile	18 (2.0%)
MUAC^b^^,^ c, cm	24.1 (23.0, 26.0)
Low MUAC (<23 cm)	344 (24.9%)

Abbreviations: GWG, gestational weight gain; IOM, Institute of Medicine; MUAC, mid‐upper arm circumference.

a Sample size per outcome: GWG, *n* = 917, (only women in their second and third trimester of gestation at Visit 1 and who completed both Visits 1 and 2 were considered).

b GWG in the second trimester, *n* = 554; GWG in the third trimester, *n* = 348; MUAC, *n* = 1,384.

c median (25th, 75th), or n (%) all such values.

### 3.2 Determinants of GWG per week and low GWG among pregnant women

Analyses performed using the minimally adjusted models indicated that two predictors were consistently and significantly associated with both GWG per week and low GWG, as defined by both U.S. IOM guidelines and INTERGROWTH‐21st standards (Table 3), and some predictors were significant in individual models.

**Table 3:** Predictors of gestational weight gain among pregnant women (minimally adjusted^a^^,^ b)

Variables	GWG (continuous) not adjusting for GA	GWG (continuous) adjusting for GA	GWG below IOM guidelines (<0.35 kg/week) adjusting for GA	GWG less than the third centile of INTERGROWTH‐21st standards not adjusting for GA	GWG <50th centile of INTERGROWTH‐21st standards not adjusting for GA
Adolescent					
Yes	−0.004 [−0.08, 0.07]	−0.01 [−0.09, 0.08]	1.4 [0.9, 2.4]	0.8 [0.5, 1.4]	1.0 [0.5, 2.0]
No	Ref	Ref	Ref	Ref	Ref
Education					
Any formal education	0.06 [−0.02, 0.14]	0.06 [−0.02, 0.14]	0.9 [0.5, 1.5]	0.5 [0.3, 0.9]*	1.1 [0.6, 1.9]
No education	Ref	Ref	Ref	Ref	Ref
Principal occupation					
Housewife	−0.06 [−0.15, 0.04]	−0.05 [−0.15, 0.04]	1.4 [0.9, 2.1]	1.0 [0.64, 1.57]	1.0 [0.7, 1.7]
Nonhousewife	Ref	Ref	Ref	Ref	Ref
Number of pregnancies	−0.009 [−0.02, 0.002]	−0.009 [−0.02, 0.001]	1.04 [0.97, 1.12]	1.1 [0.97, 1.16]	1.11 [1.03, 1.20]*
Number of living children	−0.0068[−0.02, 0.01]	−0.009 [−0.02, 0.006]	1.02 [0.94, 1.11]	1.1 [0.98, 1.20]	1.0 [0.9, 1.1]
Height	0.004 [−0.001,0.01]	0.004 [−0.001, 0.01]	0.96 [0.92, 0.98]*	0.98 [0.95, 1.02]	0.94 [0.91, 0.98]*
Household livestock index					
Above the median	0.003 [−0.05, 0.06]	0.001 [−0.06, 0.06]	1.0 [0.7, 1.4]	0.8 [0.6, 1.2]	1.1 [0.8, 1.6]
At or below the median	Ref	Ref	Ref	Ref	Ref
Housing quality index					
Above the median	−0.02 [−0.10, 0.06]	−0.02 [−0.10, 0.06]	1.1 [0.7, 1.6]	1.1 [0.8, 1.6]	1.1 [0.6, 1.8]
At or below the median	Ref	Ref	Ref	Ref	Ref
Household asset index					
Above the median	−0.02 [−0.08, 0.03]	−0.02 [−0.07, 0.04]	1.1 [0.8, 1.5]	1.1 [0.7, 1.7]	1.2 [0.8, 1.8]
At or below the median	Ref	Ref	Ref	Ref	Ref
Reported food intake					
Increased	0.004 [−0.10, 0.11]	0.01 [−0.1, 0.12]	0.9 [0.5, 1.6]	1.1 [0.6, 2.2]	1.0 [0.5, 2.0]
Decreased	−0.005 [−0.09, 0.08]	−0.004 [−0.09, 0.08]	0.8 [0.4, 1.4]	1.3 [0.7, 2.4]	0.9 [0.4, 1.8]
No change	Ref	Ref	Ref	Ref	Ref
Number of meals					
Increased	−0.03 [−0.14, 0.08]	−0.03 [−0.13, 0.08]	1.1 [0.5, 1.9]	1.2 [0.7, 2.1]	1.1 [0.5, 2.4]
Decreased	−0.02 [−0.10, 0.05]	−0.02 [−0.09, 0.05]	0.8 [0.5, 1.4]	1.2 [0.8, 2.0]	1.1 [0.6, 1.9]
No change	Ref	Ref	Ref	Ref	Ref
Levels of household food insecurity					
Food secure	−0.06 [−0.17, 0.04]	−0.07 [−0.18, 0.04]	1.8 [1.0, 3.4]*	0.20 [0.65, 2.28]	0.51 [0.84, 3.33]
Mildly food insecure	−0.03 [−0.15, 0.09]	−0.04 [−0.16, 0.09]	1.7 [0.8, 3.4]	0.10 [0.50, 2.60]	0.15 [0.49, 2.74]
Moderately food insecure	−0.04 [−0.17, 0.09]	−0.04 [−0.17, 0.09]	1.4 [0.7, 2.7]	0.03 [0.50, 2.14]	0.46 [0.79, 3.22]
Severely food insecure	Ref	Ref	Ref	Ref	Ref
Adequate minimum dietary diversity	0.01 [−0.05, 0.07]	−0.01 [−0.08, 0.05]	0.7 [0.5, 1.1]	0.93 [0.64, 1.34]	0.8 [0.5, 1.3]
Received food assistance	−0.008 [−0.10, 0.08]	−0.003 [−0.09, 0.09]	1.6 [0.9, 3.0]**	1.03 [0.57, 1.86]	1.4 [0.6, 3.4]
Haemoglobin^c^, g/dl	0.21 [0.02, 0.4]*	0.2 [0.006, 0.40]*	0.29 [0.10, 0.88]*	0.4 [0.1, 1.6]	0.9 [0.2, 4.2]
Plasma folate concentration, nmol/L	0.04 [−0.07, 0.14]	0.03 [−0.06, 0.13]	0.8 [0.4, 1.6]	0.97 [0.50, 1.90]	0.8 [0.3, 2.0]
Plasma vitamin B_12_ concentration, pmol/L	−0.04 [−0.14, 0.07]	−0.06 [−0.17, 0.05]	2.0 [0.99, 3.79]**	1.2 [0.6, 2.3]	1.4 [0.7, 2.9]
Retinol binding protein concentration^d^_,_ μmol/L	−0.07 [−0.17, 0.03]	−0.06 [−0.16, 0.04]	1.6 [0.8, 3.1]	0.97 [0.5, 2.0]	1.6 [0.6, 4.0]
Plasma ferritin concentration^d^, μg/L	−0.05 [−0.10, 0.006]	−0.05 [−0.11, 0.003]**	1.22 [0.88, 1.71]	1.5 [1.1, 2.0]*	1.1 [0.8, 1.6]
Soluble transferrin receptor concentration, mg/L	−0.05 [−0.11, 0.02]	−0.04 [−0.11, 0.03]	1.6 [1.04, 2.5]*	1.4 [0.8, 2.2]	1.0 [0.6, 1.5]
Plasma zinc concentration^d^, μg/dl	−0.002 [0.006, 0.002]	−0.002 [−0.007, 0.002]	1.0 [0.97, 1.03]	1.0 [0.98, 1.03]	1.03 [1.0, 1.07]**
α‐1‐acid glycoprotein concentration, g/L	−0.10 [−0.17, −0.02]*	−0.1 [−0.2, −0.02]*	1.7 [1.1, 2.8]*	1.9 [1,2, 3.0]*	1.3 [0.7, 2.3]
C‐reactive protein concentration, mg/L	−0.002 [−0.006, −0.002]*	−0.03 [−0.06, −0.01]*	1.14 [0.98, 1.32]	1.2 [1.02, 1.50]*	1.1 [1.0, 1.3]
Plasma histidine‐rich protein II concentration, ng/L	−0.06 [−0.13, 0.002]**	−0.06 [−0.13, 0.003]**	1.72 [1.01, 2.92]*	1.3 [0.8, 2.2]	1.2 [0.6, 2.3]

a Sample size for GWG analysis, n = 918, (only women in their second and third trimester of gestation at Visit 1 and who completed both Visits 1 and 2 were considered) and sample size for biochemistry indicators, n = 770.

b Minimally adjusted model: adjusted for year, season, and village because of the nature of the study design.

c A 0.1 g/dl in Hb concentration was associated with a 1.0‐g increase in GWG.

d Adjusted for acute phase protein.

* Statistically significant, P < .05.

** Marginally significant; P = .05–.07.

#### 3.2.1 GWG per week nonadjusting and adjusting for GA

In the analysis not adjusting for GA, increasing concentrations of AGP (*β* = −.10, 95% CI [−0.17, −0.02]) and CRP (*β* = −.002, 95% CI [−0.006, −0.002]) were associated with decreasing GWG. When adjusting for GA, this was consistent in the analysis for AGP (*β* = −.1, 95% CI [−0.2, −0.02]) and CRP (*β* = −.03, 95% CI [−0.06, −0.01]), respectively. Higher haemoglobin concentration was associated with increased GWG when not adjusting for GA (*β* = .2, 95% CI [0.02, 0.4]) and after adjusting GA (*β* = .2, 95% CI [0.006, 0.40]), respectively.

#### 3.2.2 Low GWG according to IOM guidelines, adjusting for GA

Women who reported being food secure (OR = 1.8, 95% CI [1.0, 3.4]), had higher AGP in grams per litre (OR = 1.7, 95% CI [1.1, 2.8]), higher sTfR in milligrams per litre (OR = 1.6, 95% CI [1.04, 2.5]), and higher HRP2 in nanograms per millilitre (OR = 1.72, 95% CI [1.01, 2.92]) concentration had increased odds of low GWG. Pregnant women who were taller in centimetres (OR = 0.96, 95% CI [0.92, 0.98]) and those with higher haemoglobin concentrations in grams per decilitre (OR = 0.29, 95% CI [0.10, 0.88]) had decreased odds of low GWG.

#### 3.2.3 Low GWG according to the third INTERGROWTH‐21st standards not adjusting for GA

Higher plasma ferritin in micrograms per litre (OR = 1.5, 95% CI [1.1, 2.0]), higher AGP in grams per litre (OR = 1.9, 95% CI [1.2, 3.0]), and higher CRP in milligrams per litre (OR = 1.25, 95% CI [1.02, 1.50]) concentration were associated with increased odds of low GWG. Women who had any formal education (OR = 0.5, 95% CI [0.3, 0.9]) had decreased odds of low GWG compared with those who were not formally educated.

#### 3.2.4 GWG < 50th centile of the INTERGROWTH‐21st standards not adjusting for GA

One increase in the number of pregnancies that a woman had (OR = 1.11, 95% CI, [1.03, 1.20]) was associated with increased odds of GWG below the 50th centile. Pregnant women who were taller (cm) had decreased odds of GWG below the 50th centile (OR = 0.94, 95% CI [0.91, 0.98]).

### 3.3 Determinants of low MUAC and continuous MUAC among pregnant women

The odds of low MUAC were higher among adolescent women (OR = 2.7, 95% CI [1.8, 04.0]) compared with adult women, women who identified themselves as housewives (OR = 1.97, 95% CI [1.36, 2.86]) compared with those who had other principal occupations, and among those who reported recent receipt of food assistance (OR = 1.8, 95% CI [1.04, 3.11]; Table 4). This was consistent in the analysis with continuous MUAC. One increase in the number of pregnancies that a woman had (OR = 0.90, 95% CI [0.83, 0.98]), one increase in the number of children that a woman had (OR = 0.87, 95% CI [0.78, 0.97]), greater height in centimetres (OR = 0.93, 95% CI, [0.91, 0.94]), and higher haemoglobin concentrations in grams per decilitre (OR = 0.21, 95% CI, [0.01, 0.42]) were associated with decreased odds of low MUAC. These were consistent in the analysis with continuous MUAC**.**

**Table 4:** Predictors of low mid‐upper arm circumference among pregnant women (minimally adjusted^a^^,^ b)

Variables	Minimally adjusted mean difference (95% CI)	*P* value	Minimally adjusted odds ratio or minimally adjusted mean difference(95% CI)	*P* value
Adolescent				
Yes	−1.3 [−1.7, −0.9]	<.0001	2.7 [1.8, 4.0]	<.0001
No			Ref	
Education				
Any formal education	−0.3 [−0.7, 0.2]	.25	1.5 [0.95, 2.45]	.0807
No education	Ref		Ref	
Principal occupation				
Housewife	−0.7 [−1.1, −0.3]	.001	1.97 [1.36, 2.86]	.0006
Nonhousewife	Ref		Ref	
Number of pregnancies	0.2 [0.1, 0.3]	<.0001	0.90 [0.83, 0.98]	.02
Number of living children	0.2 [0.1, 0.3]	<.0001	0.87 [0.78, 0.97]	.01
Height, cm	0.08 [0.06, 0.11]	<.0001	0.93 [0.91, 0.94]	<.0001
Household livestock index				
Above the median	0.16 (−0.17, 0.49)	.33	1.2 (0.9, 1.6)	.29
At or below the median	Ref		Ref	
Housing quality index				
Above the median	0.4 [0.06, 0.74]	.02	0.85 [0.61, 1.20]	.33
At or below the median			Ref	
Household asset index				
Above the median	0.15 [−0.1, 0.4]	.23	0.8 [0.6, 1.2]	.35
At or below the median			Ref	
Reported food intake				
Increased	−0.4 [−0.97, 0.16]	.11	1.4 [0.9, 2.2]	.09
Decreased	−0.02 [−0.52, 0.49]		0.9 [0.6, 1.4]	—
No change	Ref		Ref	
Number of meals				
Increased	−0.5 [−1.0, 0.1]	.11	1.6 [1.0, 2.6]	.06
Decreased	−0.2 [−0.6, 0.3]		1.2 [0.8, 1.8]	—
No change	Ref		Ref	
Levels of household food insecurity				
Food secure	0.2 [−0.4, 0.8]	.52	−0.1 [0.52, .147]	.91
Mildly food insecure	0.4 [−0.3, 1.1]		−0.1 [0.46, 1.91]	**—**
Moderately food insecure	0.1 [−0.4, 0.5]		0.02 [0.66, 1.58]	**—**
Severely food insecure	Ref		Ref	
Adequate dietary diversity	0.2 [−0.2, 0.6]	.28	0.9 [0.7, 1.3].	.63
Received food assistance	−0.9 [−1.6, −0.3]	.006	1.80 [1.04, 3.11]	.04
Haemoglobin, g/dl^c^	2.8 [1.4, 4.1]	<.0001	0.21 [0.01, 0.42]	.04
Plasma folate concentration, nmol/L	0.6 [−0.03, 1.26]	.06	0.6 [0.3, 1.1]	.09
Plasma vitamin B_12_ concentration, pmol/L	−0.4 [−1.0, 0.2]	.19	1.20 [0.58, 2.48]	.63
Retinol binding protein concentration^d^, μmol/L	0.26 [−0.37, 0.88]	.42	0.9 [0.5, 1.7]	.86
Plasma ferritin concentration^d^_,_ μg/L	−0.1 [−0.3, 0.2]	.57	0.9 [0.7, 1.2]	.60
Soluble transferrin receptor concentration, mg/L	−0.1 [−0.5, 0.4]	.78	1.4 [0.8, 2.3]	.23
Plasma zinc concentration^d^, μg/dl	0.02 [−0.002, 0.04]	.07	0.98 [0.96, 1.00]	.06
α‐1‐acid glycoprotein concentration, mg/L	−0.4 [−0.9, 0.2]	.20	1.7 [0.9, 3.3]	.10
C‐reactive protein concentration, g/L	−0.01 [−0.2, 0.2]	.94	1.0 [0.9, 1.2]	.78
Plasma histidine‐rich protein II concentration, ng/L	−0.2 [−0.6, 0.2]	.34	0.98 [0.69, 1.41]	.92

a Sample size for MUAC analysis, *n* = 1,384.

b Minimally adjusted model: adjusted for year, season, and village because of the nature of the study design.

c A 0.1 g/dl in Hb concentration was associated with a 1.0‐cm increase in MUAC.

d Adjusted for acute phase protein.

## 4 DISCUSSION

The prevalence of low GWG was high among pregnant women in Zinder irrespective of whether the IOM guidelines or the INTERGROWTH‐21st standards were used. More than one in two pregnant women (62.9 %) had low GWG (GWG per week <0.35 kg) according to the 2009 IOM guidelines, and more than one fourth of the pregnant women (27.4%) had low GWG (GWG per week less than the third centile) based on the INTERGROWTH‐21st standards. Similar results were reported in Ghana, where the estimated prevalence of low GWG based on the GWG over the whole pregnancy according to IOM guidelines was 62.7%, and the percentage of women with GWG less than the third centile of GWG according to the INTERGROWTH‐21st standards was 27% (Adu‐Afarwuah et al., 2017). In Ethiopia, the prevalence of low GWG based on the total GWG according to the IOM criteria was also high (69.3%; Asefa & Nemomsa, 2016). Given the adverse maternal and child health outcomes associated with low GWG, our findings indicate that low GWG is a concern in the study area and highlights the need for effective maternal health and nutrition interventions to influence these outcomes (Hamad, Cohen, & Rehkopf, 2016).

The two sets of cut‐offs used for GWG in the present study differ substantially. For example at 28 weeks of GA, the IOM cutoff for low GWG is 0.35 kg/week, and the third centile of the INTERGROWTH‐21st standard is 0.27 kg/week. Consequently, we found that the prevalence of low GWG according to the 2009 U.S. IOM guideline (GWG <0.35 kg/week) was about two times higher than the prevalence based on the third centile of the INTERGROWTH‐21st standards. The IOM guidelines were developed based on the available published literature as well as the reports of consultants, and the goal was to identify GWG values or range of GWG values in the U.S. population associated with lowest prevalence of adverse outcomes including caesarean delivery, postpartum weight retention, preterm birth, small‐ or large‐for‐GA birth, and childhood obesity (IOM and NRC, 2009). Conversely, the INTERGROWTH‐21st standards are derived from a prospective longitudinal, multicountry population study. They represent GWG of healthy, well‐nourished, and educated women who had a BMI between 18.5 and 24.9 in the first trimester of pregnancy with good maternal and perinatal outcomes (Cheikh Ismail et al., 2016). Interestingly, the INTERGROWTH project did not find any country‐specific differences, suggesting that these standards may be useful internationally. However, the third centile of the INTERGROWTH‐21st standards identifies women with severely low GWG as compared with the IOM guidelines, and using another, slightly higher centile may be more useful for identifying women at risk of low GWG.

In the present study, we were also interested in assessing the prevalence of low MUAC as another indicator of nutritional status during pregnancy, because MUAC reflects both past and current nutritional status (WHO expert Committee on physical status, 1995). We found that a significant proportion of pregnant women had low MUAC. In a study conducted in Ethiopia, a similar prevalence of low MUAC (31.8%) was reported among pregnant women (Mariyam & Dibaba, 2018), although this study applied a cutoff of 21 cm.

Our study revealed that higher concentrations of markers of systemic inflammation (AGP and CRP) and higher concentrations of sTfR (indicative of iron deficiency and/or increased erythropoiesis) were associated with decreased GWG per week and increased odds of low GWG. Inflammation and iron deficiency can be accompanied by loss of appetite, decreasing food intake, and possibly leading to low weight gain during pregnancy (Katona & Katona‐Apte, 2008; Raiten et al., 2015; Scrimshaw, 1977). However, it is interesting to note that higher plasma ferritins concentrations (indicative of iron sufficiency and/or inflammation) were associated with increased odds of GWG less than the third centile of INTERGROWTH. Although ferritin concentrations were adjusted for inflammation in the present study, it is possible that adjustments did not fully capture the effects of inflammation.

In the present study, pregnant women with higher haemoglobin concentrations had decreased odds of low GWG and decreased odds of low MUAC. Although we are unaware of previous studies that have reported direct associations between GWG and haemoglobin, previous studies have shown a similar relationship between haemoglobin concentration and MUAC (Addis Alene & Mohamed Dohe, 2014; Makhoul et al., 2012; Saaka, Oladele, Larbi, & Hoeschle‐Zeledon, 2017). Maternal anaemia (Figueiredo et al., 2018; Sukrat et al., 2013) and both anaemia and low GWG are known risk factors of LBW (Edwards et al., 1996; Hellerstedt et al., 1997; Hickey et al., 1997; Schieve et al., 1998), intrauterine growth restriction (Strauss & Dietz, 1999), and SGA (Hellerstedt et al., 1997; Hickey et al., 1997; Nielsen et al., 2006).

We found that taller women had lower odds of low GWG and lower odds of low MUAC. Similar findings were reported in the Philippines, where higher maternal height was associated with greater total weight gain (Siega‐Riz & Adair, 1993). In a study conducted in Tanzania, taller women (>159.5 cm) were also more likely to gain more weight than shorter women (<151.5 cm; Changamire et al., 2014). Maternal height reflects both genetic and environmental factors, as well as long‐term dietary intake and nutritional status (Perkins, Subramanian, Davey Smith, & Ozaltin, 2016), all of which have been also shown to influence GWG (IOM and NRC, 2009).

Increased gravidity was associated with increased odds of low GWG (<50th centile); similar findings have been reported in Tanzania (Changamire et al., 2014). It is possible that higher gravidity and parity, and frequent reproductive cycling, may result in maternal nutritional depletion in the context of high food insecurity and thus increasing the risk of SGA and LBW (Klerman, Cliver, & Goldenberg, 1998; Miller, 1991). However, increases in the number of pregnancies and children were associated with decreased odds of low MUAC. MUAC has been shown to be relatively stable throughout pregnancy (WHO expert Committee on physical status, 1995), except in adolescence; thus, the relationship between gravidity and MUAC may be mediated by age, reflecting both lower gravidity and increased odds of low MUAC among adolescents.

Pregnant adolescents had higher odds of low MUAC. This is consistent with the findings in Nepal and Malawi, where lower age was associated with decreased MUAC during pregnancy (Chithambo, May May 2017; Ghosh et al., 2017). Adolescence is a period of rapid growth, and when pregnancy occurs during this period, there is increased competition for nutrients with the fetus, which increases the risk of undernutrition among adolescent pregnant women (Das et al., 2017).

We found that pregnant women who reported food assistance during the present pregnancy had decreased MUAC and higher odds of low MUAC. Similar results on the relationship between food assistance and low MUAC have been reported in Ethiopia (Gebre, Biadgilign, Taddese, Legesse, & Letebo, 2018). Somewhat unexpectedly, however, we observed that women in food secure households had increased odds of GWG below IOM guidelines. It is possible that dietary patterns differed between women in food secure versus insecure households. Outside of economic constraints, it has been shown that food intake during pregnancy is largely driven by personal preferences and cravings, cultural beliefs, food taboos (i.e., prohibition against consuming certain foods), and beliefs surrounding pregnancy physiology (Kavle & Landry, 2018).

To our knowledge, this is the first study to examine GWG and undernutrition in pregnant woman in Niger and its determinants. The present study has several strengths. Due to the extensive data collected by highly trained and supervised field workers, we were able to examine numerous potential predictors of GWG and MUAC. All data were collected over >12 months allowing us to account for the effect of season on outcomes. Despite its strengths, this study has several limitations. First, the present study was limited to two health districts, and the findings are thus not representative of the population of the entire Zinder region, nor of Niger as a whole. Second, we performed analysis only for complete cases. This method reduces the sample size and may lead to loss of power to detect a significant association. Third, GWG assessment was based on only two weight measurements taken 1 month apart; longer term observation may more accurately describe the pattern of the true GWG of these women. Fourth, we classified pregnant women according to the IOM guidelines for GWG, which are based on pre‐pregnancy BMI, data which were not available in the present study. In addition, the INTERGROWTH‐21st standards are derived from healthy, well‐nourished, and educated women, and participants had normal BMI in the first trimester of pregnancy (Cheikh Ismail et al., 2016). Thus, it is likely that some women in the present study were misclassified according to the IOM guidelines and the INTERGROWTH‐21st standards. Nevertheless, by using both sets of cutoffs and running analyses with multiple definitions of low GWG, we were likely able to identify women at highest risk of low GWG and explore risk factors associated with low GWG. Lastly, a significant proportion of pregnant women in the study population (16.3%) were adolescent, but due to the lack of a specific MUAC recommendation for the adolescent age group, those women were classified using the same guidelines and standards as adult women. For GWG, the IOM did not see sufficient evidence to support a specific guideline for adolescents (IOM and NRC, 2009). Similarly, we did not find a higher risk of low GWG among adolescent in the present study, but further research is needed (Harper, Chang, & Macones, 2011).

## 5 CONCLUSION

The prevalence of low GWG was high among pregnant women in the Zinder region of Niger, and the odds of low GWG were consistently associated with higher concentrations of markers of inflammation (AGP and CRP). A considerable proportion of pregnant women also had low MUAC. Considering the importance of adequate GWG and MUAC for maternal health and pregnancy outcomes, effective and cost‐effective interventions (e.g., daily iron folic acid supplementation; Baltussen, Knai, & Sharan, 2004, behaviour change communication about nutrition; Lamstein et al., 2014, and balanced protein energy dietary supplementation; Imdad & Bhutta, 2012) to ensure adequate GWG and nutritional status should be considered.

## CONFLICTS OF INTEREST

The authors declare that they have no conflicts of interest.

## CONTRIBUTIONS

The NiMaNu Project was conceived and designed by SYH, KRW, and CTO. Data were analysed by CTO with the guidance from RRY and SYH. Results were interpreted by all co‐authors. CTO drafted the manuscript, and SYH edited the manuscript. All authors reviewed and approved the final version of the manuscript.

## Supporting information


**Figure S1**:
Study participants' GWG per week compared to the INTERGROWTH‐21th standards
