# Supplementary material for: Prevalence and determinants of gestational weight gain among pregnant women in Niger
Source: Matern Child Nutr. 2019 Sep 30;16(1):e12887. doi: 10.1111/mcn.12887 (PMC7038899; doi:10.1111/mcn.12887)
Supplement: Supplementary file 1 — Figure S1: Study participants' GWG per week compared to the INTERGROWTH‐21th standards [file MCN-16-e12887-s001.docx]

**Supplementary Figure 1**: Study participants’ GWG per week compared to the INTERGROWTH-21th standards
